# Identification of a Cardiac Specific Protein Transduction Domain by *In Vivo* Biopanning Using a M13 Phage Peptide Display Library in Mice

**DOI:** 10.1371/journal.pone.0012252

**Published:** 2010-08-17

**Authors:** Maliha Zahid, Brett E. Phillips, Sean M. Albers, Nick Giannoukakis, Simon C. Watkins, Paul D. Robbins

**Affiliations:** 1 Department of Microbiology and Molecular Genetics, University of Pittsburgh School of Medicine, Pittsburgh, Pennsylvania, United States of America; 2 Department of Pathology, University of Pittsburgh School of Medicine, Pittsburgh, Pennsylvania, United States of America; 3 Center for Biologic Imaging, University of Pittsburgh School of Medicine, Pittsburgh, Pennsylvania, United States of America; University of Delhi, India

## Abstract

**Background:**

A peptide able to transduce cardiac tissue specifically, delivering cargoes to the heart, would be of significant therapeutic potential for delivery of small molecules, proteins and nucleic acids. In order to identify peptide(s) able to transduce heart tissue, biopanning was performed in cell culture and *in vivo* with a M13 phage peptide display library.

**Methods and Results:**

A cardiomyoblast cell line, H9C2, was incubated with a M13 phage 12 amino acid peptide display library. Internalized phage was recovered, amplified and then subjected to a total of three rounds of *in vivo* biopanning where infectious phage was isolated from cardiac tissue following intravenous injection. After the third round, 60% of sequenced plaques carried the peptide sequence APWHLSSQYSRT, termed cardiac targeting peptide (CTP). We demonstrate that CTP was able to transduce cardiomyocytes functionally in culture in a concentration and cell-type dependent manner. Mice injected with CTP showed significant transduction of heart tissue with minimal uptake by lung and kidney capillaries, and no uptake in liver, skeletal muscle, spleen or brain. The level of heart transduction by CTP also was greater than with a cationic transduction domain.

**Conclusions:**

Biopanning using a peptide phage display library identified a peptide able to transduce heart tissue *in vivo* efficiently and specifically. CTP could be used to deliver therapeutic peptides, proteins and nucleic acid specifically to the heart.

## Introduction

Ischemic heart disease and occlusive coronary artery disease continue to be the number one killer in the developed world. There are an estimated 500,000 acute ST-elevation myocardial infarctions (MI) in the US alone each year [1], and this is becoming an increasingly significant problem in the developing world [2]. Current approaches for management of an acutely occluded coronary artery leading to an MI consist of anti-platelet and anti-thrombotic strategies with intervention aimed at opening the infarct-related artery in a timely fashion. Although this approach is able to protect cardiomyocytes from necrosis, with resulting decrease in morbidity and mortality, it necessitates exposing the heart to post-ischemic reperfusion injury. Limiting this reperfusion injury and decreasing apoptosis would ultimately lead to greater myocardial salvage and prevention of development of heart failure.

Numerous animal studies have identified biological agents able to ameliorate this ischemia-reperfusion injury and reduce the ultimate infarct size [3], [4]. However, further development of these approaches is hindered by the inability to deliver the biologic agents to the myocardium in a tissue-specific, efficient and rapid manner. A protein transduction peptide specific for the heart would be able to deliver biologic agents in a timely fashion to the heart when given at the time of reperfusion for an infarction.

Protein transduction domains (PTD) are small cationic peptides that can cross cellular membranes, and are able to transport large, biologically active molecules into mammalian cells in culture as well as *in vivo*. The limitation of PTDs is the non-specific transduction of all tissue types with some tissues, such as liver and kidney, taking up the PTD much more avidly than heart tissue. Thus there is a need to identify peptides able to target cardiac tissue specifically for delivery of biologics of therapeutic potential.

Screening approaches using peptide phage display libraries are effective for identifying peptides able to bind to specific ligand targets as well as identifying peptides with novel properties. Phage display uses filamentous bacteriophage, such as M13, that are able to replicate in E. coli. The proteins or peptides to be displayed are fused to the N-terminus of phage coat protein pIII or pVIII and thus are present on the surface of the phage. Screening of peptide phage display libraries has been used *in vivo* to identify peptides able to target tumor vasculature [5], adipose tissue [6] and pancreatic islet cells [7]. In addition, it has been used to identify peptides able to facilitate internalization of intact, infectious phage into specific cell types such as synovial fibroblasts [8]. *In vivo* phage display also has been utilized to target atherosclerotic plaques [9], and to probe the heart vasculature for endothelial markers [10]. Although *in vitro* selection of a specific peptide sequence carrying phage resulted in increased targeting of cardiomyocytes by phage *in vivo* [11], it remains to be determined if the peptide can actually deliver “cargo” peptides or proteins of therapeutic potential to the heart. If such were indeed the case, it would open up new avenues of drug development, leading to delivery of therapeutics directly to the ischemic heart.

In the current study, we utilized a combinatorial approach of cell culture and *in vivo* biopanning using an M13 phage peptide display library to identify peptide(s) with potential for cardiomyocyte transduction *in vivo* in a tissue specific manner. We have identified a peptide, termed *C*ardiac *T*argeting *P*eptide or CTP that is able to transduce cardiomyocytes specifically in culture and *in vivo*. This peptide could be used to deliver peptides, proteins or nucleic acid of therapeutic potential specifically to the heart.

## Methods

### Phage display

A combined approach of *in vitro* and *in vivo* screening of a phage peptide display library for cardiomyocyte-specific transduction peptides was utilized. Cardiomyoblasts, H9C2 cells (ATCC, CRL-1446), were incubated with 10 ul (1×10^11^ pfu) of a 12-mer M13 phage peptide display library (NEB, E8110S), for 6 hours at 37°C, 5% CO_2_. Cells were then washed extensively, trypsinized and lysed by a single freeze-thaw cycle. Recovered phage was tittered and amplified. The post-amplified phage was again tittered and administered intravenously by retro-orbital injection at a dose of 3.5×10^11^, to a female Balb/c mouse. The mice were pre-treated with intra-peritoneal injection of Chloroquine (20 mg/Kg) 24 hours prior to and on the day of the phage injection, in order to minimize intra-lysosomal destruction of internalized phage and increase the chances of recovering internalized phage. The phage was allowed to circulate for 24 hours, after which the mice were euthanized and heart and kidney tissues obtained. The rationale for this approach was based on the observation that after intravenous injection, native M13 phage had a half-life in blood of 4.5 hours [12]. Therefore we allowed the phage to circulate for ∼5–6 half-lives to maximize the chance of uptake by cardiomyocytes and minimize contamination with non-specific phage circulating in the blood stream. To minimize the destruction of internalized phage in lysosomal compartments, the mice were pre-treated with Chloroquine, a drug known to increase the pH of lysosomal compartments and theoretically decrease intracellular destruction of phage. The collected tissues were digested with collagenase and phage recovered by a single freeze/thaw cycle. Recovered phage was then tittered, normalized by tissue weight and subsequently amplified for a second round of biopanning. A total of three *in vivo* biopanning rounds were performed followed by sequencing of 10 plaques. All animal studies were approved by the University of Pittsburgh Institutional Animal Care and Use Committee (protocol approval number 0804422A-1).

### Confocal microscopy

The cardiac targeting peptide (CTP) was synthesized in the University of Pittsburgh Peptide Synthesis Facility in either 6-carboxyfluorescene (CTP-6CF) labeled or biotinylated forms or conjugated to NBD (Nemo-binding domain), an 11-amino acid peptide (TALDWSWLQTE) which inhibits activation of the inducible NF-κB Kinase (IKK) by binding to the regulatory subunit (Nemo) of IKK.

A cardiomyoblast cell line (H9C2; ATCC, CRL-1446), a mouse fibroblast cell line (NIH/3T3; ATCC, CRL-1658), a murine fibrosarcoma cell line (C57/BL6 derived fibrosarcoma MCA-205 cell line, kindly provided by P. H. Basse, Dept. of Immunology, University of Pittsburgh, Pittsburgh, PA), a cervical cancer cell line (HeLa; ATCC, CCL-2), and a human kidney tubular cell line (HK-2; ATCC, CRL-2190) were incubated with increasing concentrations of CTP-6CF for 30 minutes. Cells were then washed 6 times with PBS, fixed with 2% Paraformaldehyde, and counter-stained with DRAQ5 (Molecular Probes, F1303), a nuclear stain. Cells were examined by confocal fluorescent microscopy.

### Luciferase assays

H9C2 cells and MCA205 cells were transfected using Lipofectamine (Invitrogen, 11668-027) with a reporter plasmid expressing luciferase under an NF-κB promoter site as well as a Renilla control plasmid for normalization of transfection efficiencies. Twenty-four hours later, cells were treated with increasing concentrations of CTP-NBD and 30-minutes later challenged with murine TNF-α, 10 ng/ml, for 3 hours. Cells were then washed, trypsinized, lysed and supernatant collected for Luciferase activity assay. Differences across groups were compared using an unpaired Student's t-test. A two-tailed p-value of <0.05 was considered statistically significant.

### *In vivo* imaging studies

The initial *in vivo* targeting studies were performed using CTP-6CF. Female Balb/C mice were injected retro-orbitally with CTP-6CF (25 mg/Kg) and euthanized 15 minutes later. Heart cross-sections were stained for actin using phalloidin Alexa- 647 (Molecular Probes, A22287) and stained for laminin using a rabbit anti-laminin antibody followed by a goat anti-rabbit Cy3 (Jackson ImmunoResearch, 111-167-003) secondary antibody. Five non-overlapping sections were taken from each heart for quantification of green fluorescence (CTP-6CF) expressed as a percentage of total area (blue; stained for actin).

A control peptide (CON; ARPLEHGSDKAT), picked from the original, unselected M13 phage library, CTP and 8-Lysine (8K, a homopolymer of lysine), a known cationic protein transduction domain, were synthesized in a biotinylated form. 200 µM of biotinylated CTP, CON and 8K, or equivalent volume of PBS, were incubated with 10 ul of Streptavidin-Alexa488 (2 ng/ml; Molecular Probes, S32354) for 2 hours at room temperature. Female Balb/c mice were intravenously (retro-orbitally) injected with peptides at a dose of 10 mg/Kg and then euthanized 30 minutes post-injection. Mice were also injected with biotinylated CTP conjugated to Streptavidin-Alexa488 at a dose of 10 mg/Kg and euthanized after varying circulation times to allow for tracking studies to be performed. Post-euthanasia heart, liver, lung, spleen, kidney, skeletal muscle and brain were harvested for cryosectioning followed by confocal microscopy. Sections were cross-stained with DRAQ5, a nuclear stain. For confocal microscopy, laser intensities/gains were set using negative control (PBS injected) heart tissue to minimize background fluorescence. Once the laser intensity for FITC was set using the control hearts from PBS-injected mice, it was kept constant across all subsequent imaging. Also serial scanning was performed to prevent “bleed-through” from one laser wavelength to another.

Biotinylated CON or CTP peptides were labeled with neutravidin-conjugated fluospheres (Molecular Probes, F8770) with an overnight incubation at 4°C. These fluospheres are 40 nm in diameter and fluoresce at an excitation wavelength of 605 nm, allowing for *in vivo* bead tracking. Female Balb/c mice received intracardiac injections of fluospheres-labeled CON peptide, CTP peptide or control PBS incubated fluospheres alone. Mice were anesthetized with isoflurane delivered by the XGI-8 Gas Anesthesia System (Xenogen). Initial isoflurane concentration was set to 2.5% and was reduced to 1.5% once the animals were anesthetized. Mice were then imaged at 30, 60, 120, and 180 minutes post-injection with the IVIS Lumina (Caliper Life Sciences Inc.). All mouse studies were approved by the Institutional Animal Care and Use Committee at the University of Pittsburgh (protocol approval number 0804422A-1).

## Results

### Identification of a cardiac specific transduction peptide by biopanning of a M13 phage display peptide library

**Figure 1:**
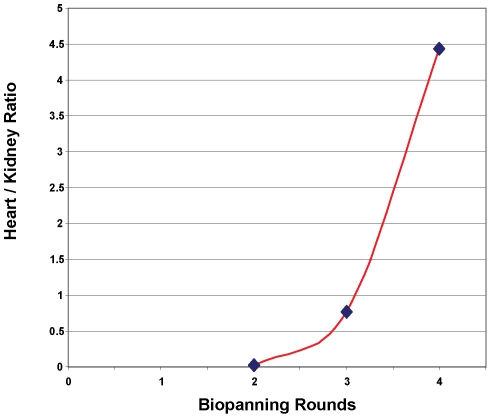
Enrichment of cardiac specific phage by multiple rounds of biopanning. After a single, screening cycle of phage incubated with H9C2 cells, recovered phage was amplified, tittered and injected intravenously into Balb/c mice. After a circulation time of 24 hours, mice were euthanized, heart and kidney dissected, digested with collagenase II, cells lysed and recovered phage tittered. Recovered phage was amplified, re-tittered and injected for subsequent round of biopanning. Phage recovered from heart versus kidney from each cycle of *in vivo* phage display was normalized by gram of tissue weight and expressed as a ratio of heart to kidney.

In order to identify a peptide able to preferentially transduce cardiac tissue *in vivo*, a screening protocol using a 12 amino acid peptide M13 peptide phage display library was utilized. The first cycle of screening of the phage peptide display library for cardiomyocytes specific transduction peptides was performed on a rat cardiomyocyte cell line, H9C2, in culture. The H9C2 cells were incubated with the M13 phage peptide display library then washed extensively and possibly internalized phage recovered following trypsinization and lysis by freeze-thaw. For each of the subsequent three rounds, the phage were injected intravenously and mice euthanized 24 hours post-injection. The hearts and kidneys were isolated, enyzmatically digested and associated phage recovered. The isolated phage were quantified and expressed as number of phage per gram of tissue weight. Following each round of *in vivo* screening, there was a steady increase in the ratio of phage recovered from the heart relative to the kidneys, suggesting enrichment of phage targeting the heart (Fig. 1). After the third round of *in vivo* screening, 10 plaques were selected and sequenced. Six of the 10 phage contained the identical nucleic acid sequence of *gcgccgtggcatctttcgtcgcagtattctcgtact* , corresponding to the peptide APWHLSSQYSRT, termed cardiac targeting peptide (CTP). A BLAST search in the NCBI database revealed that this sequence shared no homology with known naturally occurring peptides or proteins.

### Confocal microscopy analysis demonstrates preferential targeting of cardiomyoblasts

**Figure 2:**
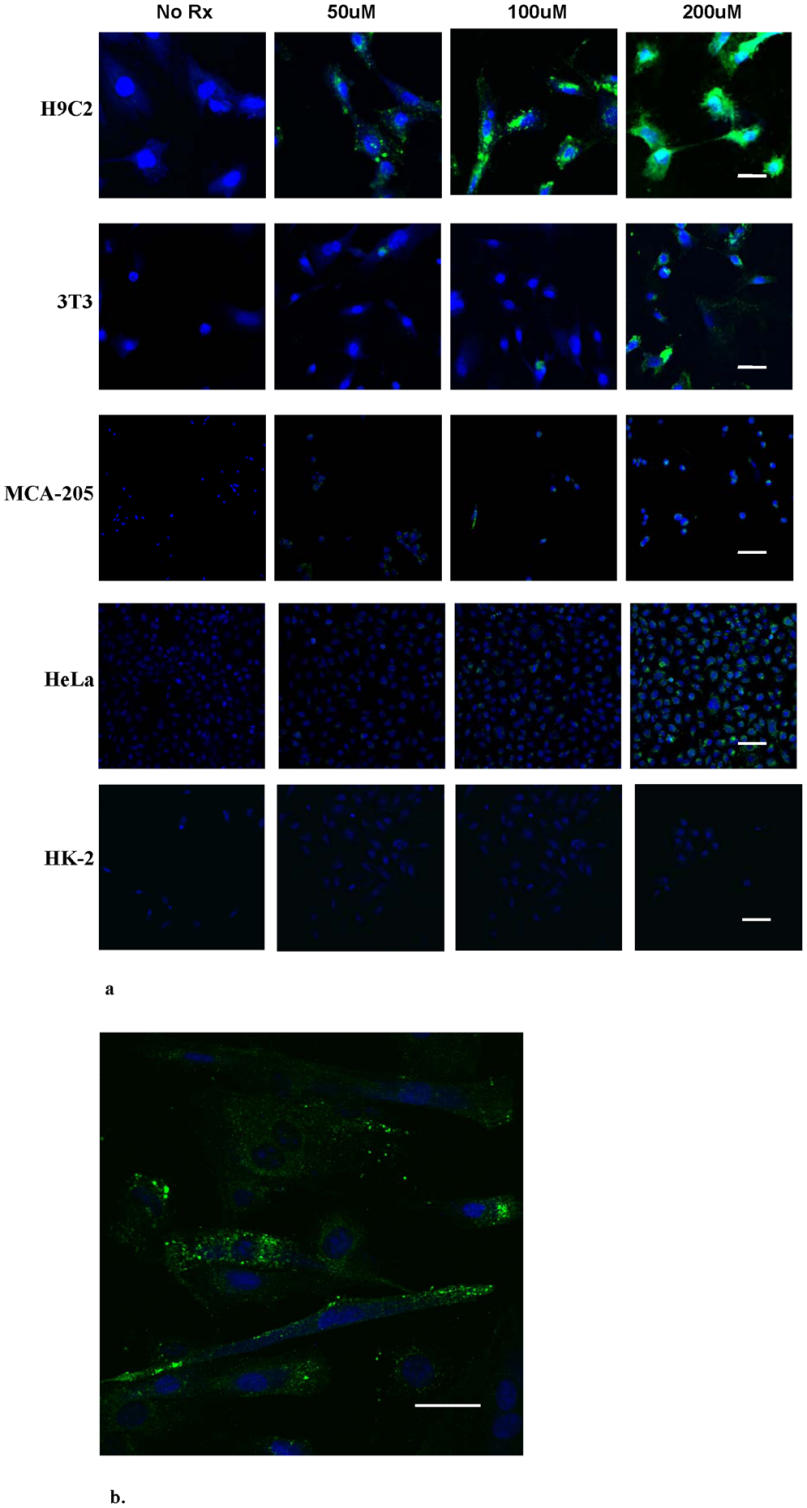
CTP specifically transduces cardiomyocytes in culture. Confocal micrographs of H9C2, 3T3, MCA-205, HeLa and HK-2 cells incubated with increasing concentrations (50 uM, 100 uM and 200 uM) of CTP-6CF for 30 minutes, followed by multiple washings and cross-staining with a nuclear stain, DRAQ5 (20×) (a). A higher (40×) confocal micrograph of H9C2 cells is shown demonstrating pattern of transduction (b); CTP-6CF - green, Nuclei – blue. Scale bars represent 100 uM.

In order to examine the ability of CTP to transduce cardiomyocytes preferentially in a dose-dependent manner, fluorescent confocal microscopy was performed using the peptide coupled to 6-carboxyflouroscene (6-CF). H9C2, 3T3, MCA-205, HeLa and HK-2 cells were incubated with increasing concentrations of CTP-6-CF, washed, fixed and counterstained with DRAQ5, a nuclear stain. As shown in figure 2, significant internalization of CTP-6-CF was observed in H9C2 cells compared to relatively minor internalization by 3T3, MCA-205 and HeLa cells at high concentrations, with no appreciable uptake by HK-2 cells. These results, performed by confocal analysis, demonstrate both the specificity of transduction by CTP as well as that the peptide is internalized, and not simply binding to the cell membrane.

### Inhibition of IKK/NF-κB signal transduction by a CTP-NBD fusion peptide demonstrates functional delivery to cardiomyoblasts

**Figure 3:**
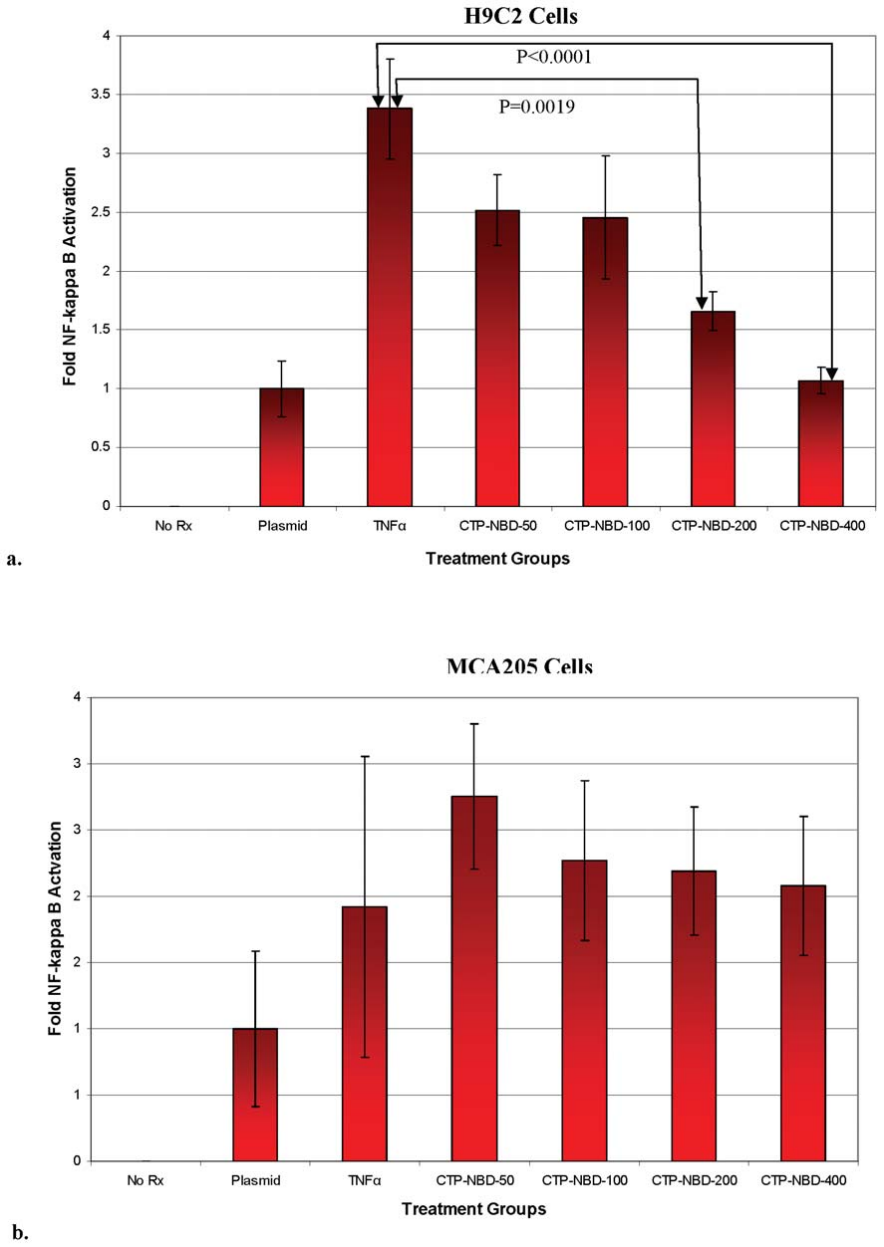
CTP functionally delivers the IKK/NF-kB inhibitory peptide NBD to cardiomyocytes. H9C2 cells (a) and MCA205 (b) cells, were co-transfected with a NF-κB dependent luciferase reporter plasmid and a control renilla-expression plasmid. Twenty-four hours post-transfection, cells were stimulated with TNF-α (10 ng/ml) or pre-treated with increasing concentrations of CTP-NBD (50 uM, 100 uM, 200 uM and 400 uM) for 30 minutes followed by TNF-α stimulation for three hours. Cells were then washed, lysed and luciferase activity measured and normalized to renilla activity. (N = 4 in each group; error bars represent one standard deviation).

To confirm functional transduction of H9C2 cells by CTP, the ability to deliver a peptide, NBD, able to block activation of the IKK/NF-κB transduction pathway, was examined. H9C2 and MCA205 cells were transfected with a plasmid expressing the luciferase marker under the control of a NF-κB-dependent promoter. Twenty-four hours post-transfection, cells were pre-treated with the CTP-NBD fusion peptide for 30 minutes, followed by stimulation with murine TNF-α for three hours. TNF-α treatment alone caused an increase in NF-κB transcriptional activity, which was inhibited by pre-treating the H9C2 cells with increasing concentrations of CTP-NBD, in a dose-dependent fashion (Fig. 3-a). In contrast, experiments performed using MCA205 cells did not show any inhibition of TNFα mediated NF-κB activation (Fig. 3b).

### CTP transduces cardiac tissue *in vivo*

**Figure 4:**
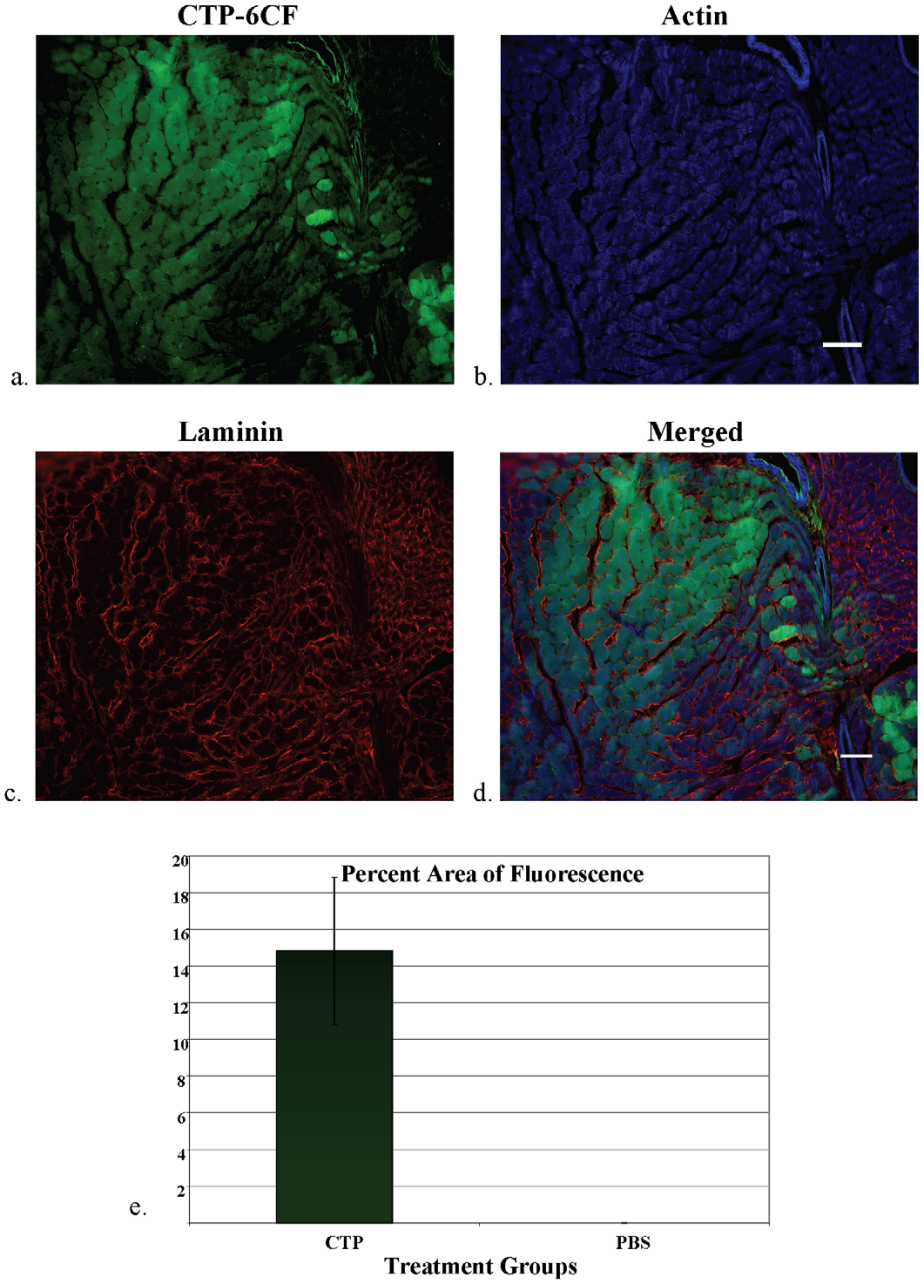
Internalization and quantification of transduction by CTP-6CF in cardiac tissue *in vivo*. Cross-sections of mouse heart were stained for actin (blue) and laminin (red). Confocal microscopy showed co-localization of CTP-6CF (a) with actin (b) but not laminin (c) as seen in the color-merged micrograph (d). FITC fluorescence from non-overlapping heart micrographs from mice injected with CTP-6CF (n = 3) or PBS (n = 3) was quantified and expressed as a percentage of total area calculated from staining for actin (Fig. 4e; error bars represent standard error of the mean). CTP-6CF – green; Actin – blue; Laminin – red. Scale bars represent 100 uM.

To demonstrate transduction of heart tissue *in vivo*, CTP-6CF or the biotinylated forms of CTP, RAN and 8K peptides coupled to Streptavidin-Alexa 488 (SA488) were injected intravenously (retro-orbitally). Mice were euthanized at varying time points and heart and multiple other organs harvested for confocal microscopy. Confocal microscopy of heart tissue from mice injected with CTP-6CF showed rapid (15 minutes) transduction of heart tissue (Fig. 4). Staining for actin and laminin showed co-localization of CTP-6CF fluorescence (green; Figure 4a) with actin (blue; Figure 4b), but not laminin (red; Figure 4c). These co-localization studies strongly suggest that CTP is internalized into cardiac cells *in vivo*, similar to the cell culture experiments. Quantification of transduction, using Metamorph software, revealed that approximately 15% of the total heart was being transduced by CTP following intravenous injection (Fig. 4e).

**Figure 5:**
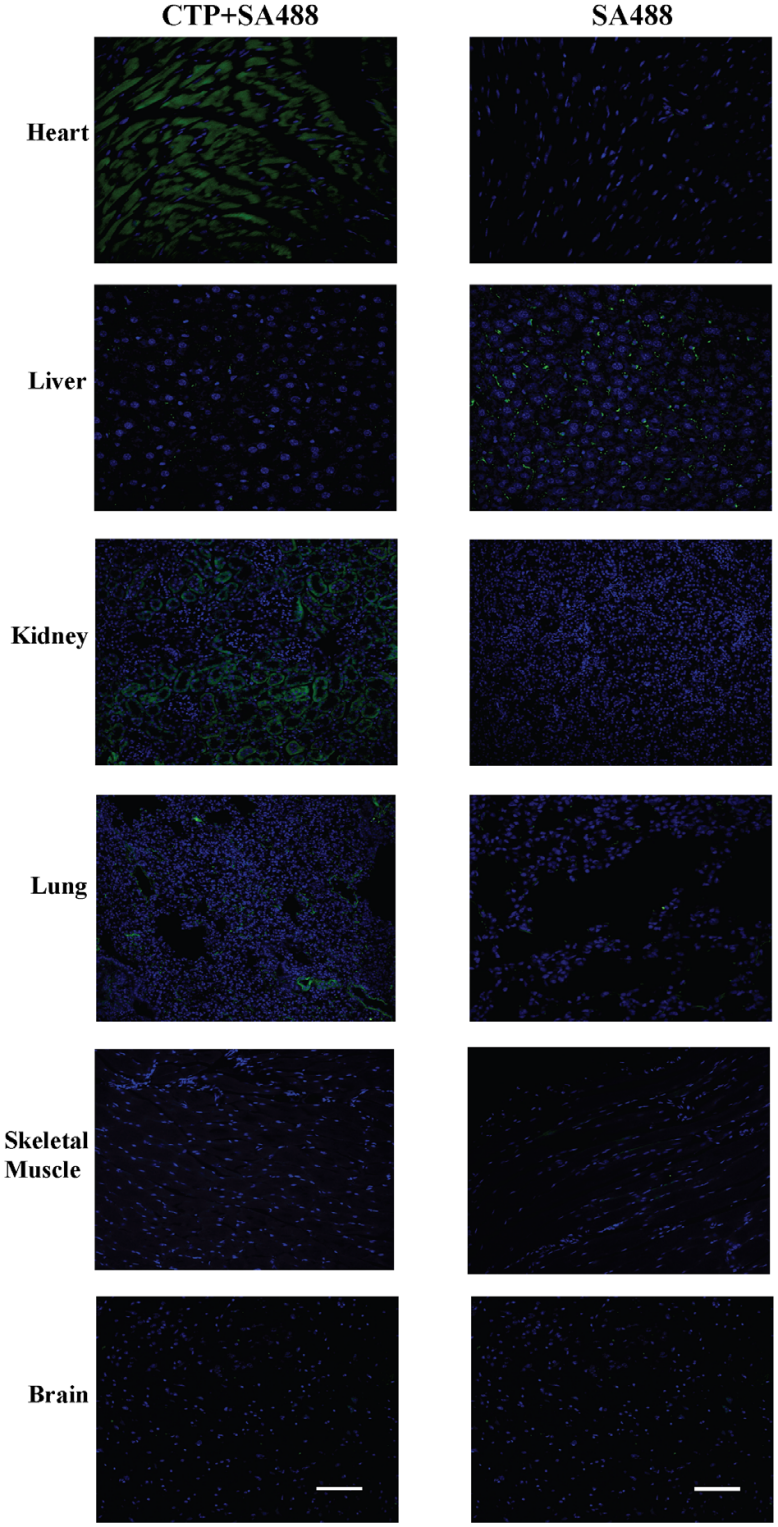
CTP specifically transduces cardiac tissue *in vivo*. Confocal analysis (20×) was performed on tissues from heart, liver, kidney, lung, skeletal muscle and brain (a–f) from mice euthanized 30 minutes after intravenous injection of CTP-biotin-SA488 conjugate (10 mg/Kg) or PBS+SA488. Slides were counter-stained with DRAQ5, a nuclear stain. CTP-SA488 – green, Nuclei – blue. N = 3 in each group; scale bars represent 100 uM.

Injection of the CTP-biotin-SA488 complex showed rapid, efficient and specific transduction of heart tissue at 30 minutes in a diffuse pattern compared to Streptavidin-Alexa 488 alone. There was no appreciable transduction seen of liver, skeletal muscle, brain (Fig. 5) or spleen (data not shown). The only other organs with uptake were a small percentage of lung capillaries as well as limited transduction of endothelial cells of the glomerular capillaries in the cortex of the kidneys (Fig. 5). These results demonstrate the specificity of CTP transduction *in vivo*.

**Figure 6:**
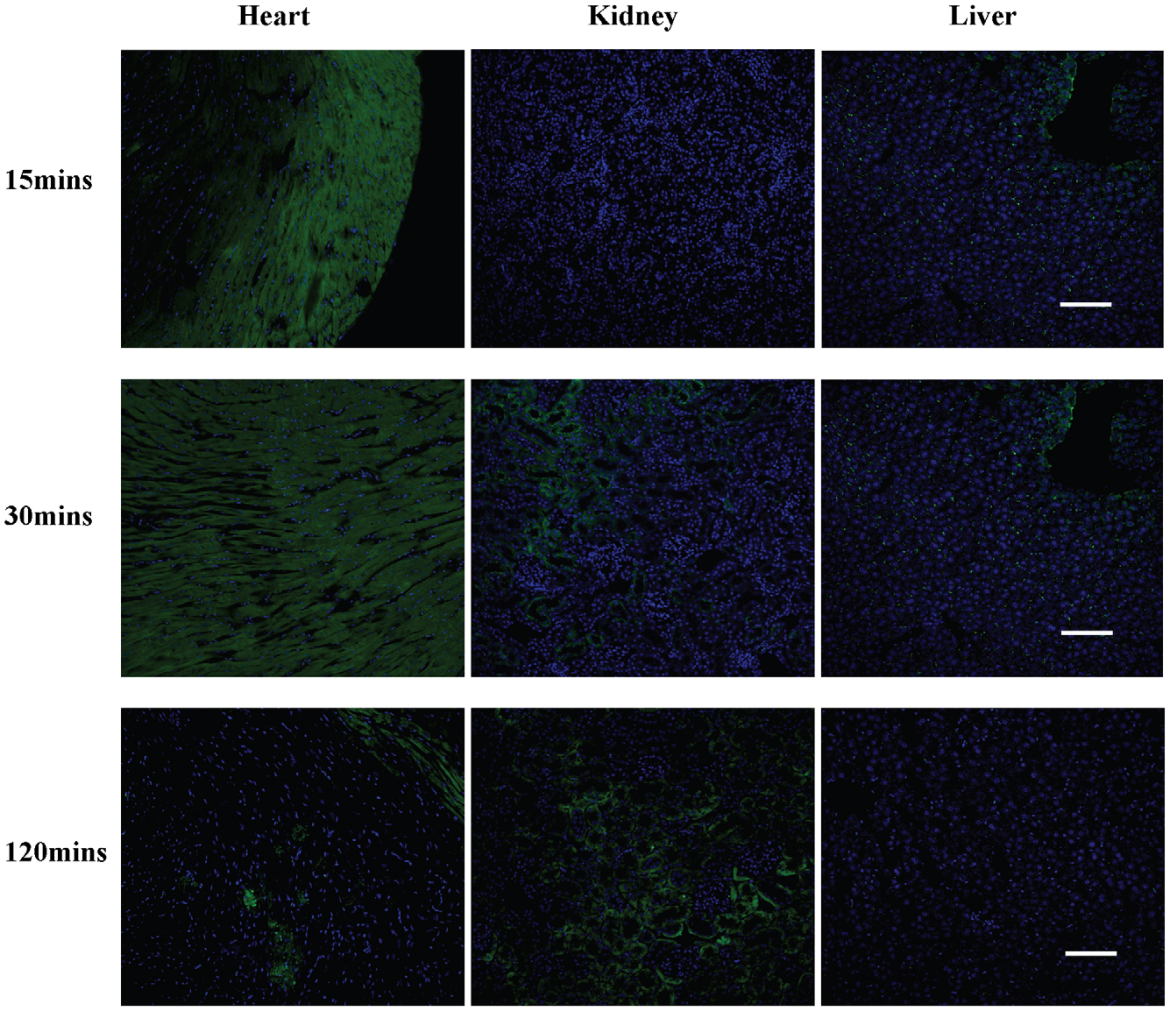
Pattern of distribution of CTP-biotin-SA488 over time *in vivo*. Mice were injected intravenously with CTP-biotin-SA488 (10 mg/Kg) and euthanized 15, 30 or 120 minutes post-injection. Heart, kidneys and liver were cross-sectioned, counter-stained with DRAQ5 and confocal microscopy performed. N = 1 in each group; scale bars represent 100 uM.

To examine the biodistribution of CTP-biotin-SA488 over time, mice were euthanized at different time points following intravenous injection. Even with the large CTP-biotin-SA488 complex, efficient transduction of the heart was seen at 15 minutes, mainly confined to the sub-epicardial region of the heart. At 30 minutes this became more diffuse and by 120 minutes there was almost no fluorescence seen in the heart (Fig. 6). Over these three time points, the fluorescence gradually increased in the kidney glomerular capillaries (Fig. 6, center column), suggesting that this might be the mode of excretion of this peptide or at least the fluorescence after peptide breakdown.

**Figure 7:**
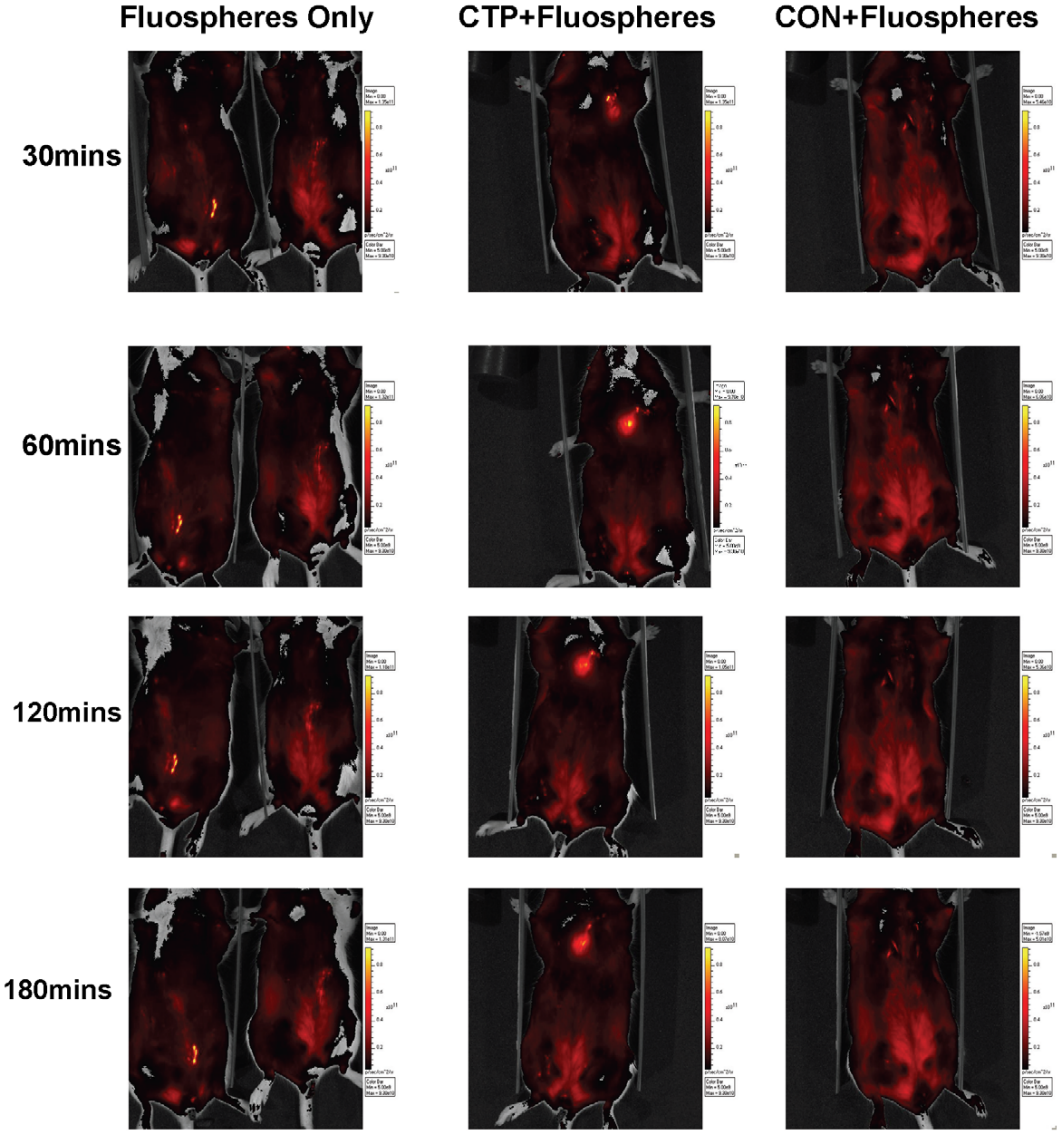
CTP targets fluospheres to the heart. Whole mouse i*n vivo* imaging was performed following intra-cardiac injection of fluospheres alone, CTP+fluospheres and RAN+fluospheres at 30, 60, 120 and 180 minutes, at a dose of 10 mg/Kg of body weight (for peptides). (N = 3 in each group).

To confirm further the ability of CTP to transduce cardiac tissue *in vivo*, the peptide was coupled to fluospheres that allow for analysis of localization by whole animal imaging. Balb/c mice were injected intracardiac with 40 nm neutravidin-labeled fluospheres alone, CTP-biotin and CON-biotin labeled with these fluospheres. Mouse imaging was performed at baseline and 30, 60, 120 and 180 minutes. CTP+fluospheres were retained in the heart, as opposed to fluospheres alone or CON+fluospheres, which dissipated immediately after injection. CTP+fluospheres could still be found localized to the heart at 3 hours post-injection (Fig. 7).

**Figure 8:**
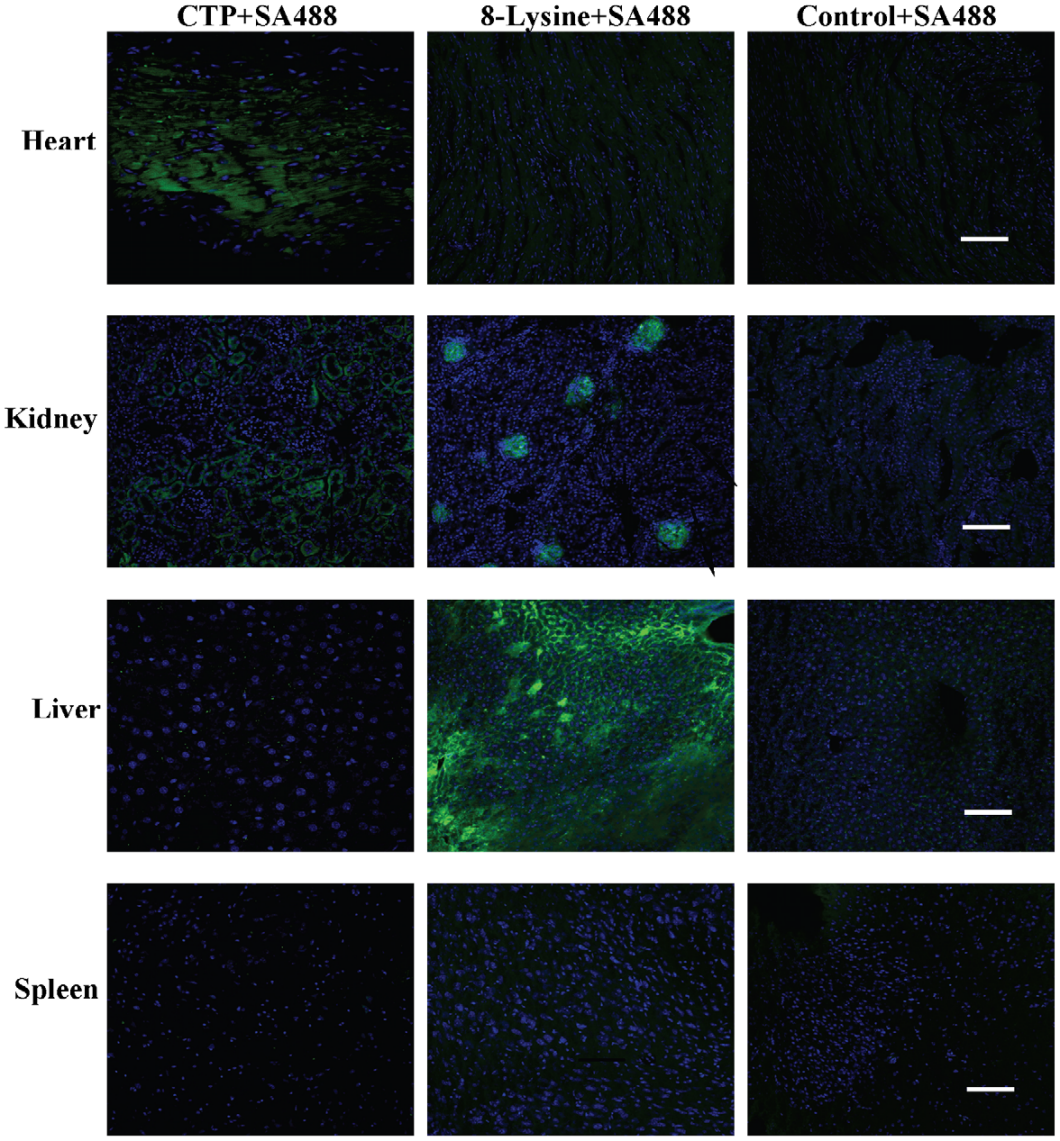
CTP specifically transduces cardiac tissue in contrast to a cationic transduction domain. Confocal analysis of heart, kidney, liver and spleen (a–d) 30 minutes following intravenous injection of CTP+SA488, 8K+SA488 or RAN+SA488 (10 mg/Kg body weight) conjugates. N = 2 in each group; scale bars represent 100 uM.

To determine the relative efficiency as well as specificity of transduction of cardiac tissue by CTP, the transduction ability of CTP was compared with 8K, a well characterized cationic protein transduction domain. Mice were injected with 10 mg/Kg dose of either CTP-SA488, 8K-SA488 or CON-SA488 conjugate and euthanized 30 minutes post-injection (Fig. 8). Mice treated with 8K-SA488 showed robust transduction of hepatocytes as well as kidney glomeruli with very little uptake in heart tissue. In contrast, CTP-SA488 conjugate showed only robust transduction of heart tissue with some uptake in the kidney glomerular capillaries and none by liver or spleen. The CON-SA488 complex did not show appreciable uptake in any organ.

It is important to note that all of the analysis of CTP transduction *in vivo* was performed with the L-form, the naturally occurring form, of the peptide. Preliminary experiments using a non-degradable D-form have shown a far more efficient transduction that persists for extended periods of time (data not shown). Thus, it appears that there is degradation of the L-CTP complexes over time.

## Discussion

The clinical application of potentially effective biological therapies for common acute cardiac conditions, like myocardial infarction, has been limited by efficiency and specificity of delivery of therapeutic agents. For example, for gene therapy approaches, such as plasmid DNA, delivery to the heart is very inefficient whereas there are significant time delays associated with cardiac gene delivery using viral-based vectors. In addition, there are issues regarding the presence of pre-existing neutralizing antibodies or immune responses to certain viral vectors. The well-characterized cell penetrating peptides, like TAT from HIV coat protein, homopolymers of arginine or lysine, are not cell specific and transduce hepatocytes and multiple other organs in addition to the heart. Therefore, identifying a peptide with transduction capabilities specific for the heart would allow for new approaches for effective cardiac delivery of therapeutics.

We previously have reported the ability to identify a synovial specific transduction peptide by screening an M13 phage peptide display library for internalized phage [8]. Thus we screened a large phage peptide display library in order to identify novel peptides potentially able to transduce cardiomyocytes *in vivo*. Indeed, we report here the identification of a specific peptide, termed CTP, which 15 or 30 minutes post-peripheral intravenous injection can efficiently and specifically transduce cardiac tissue (Fig. 4 and 5 respectively). Transduction of cardiomyocytes on confocal microscopy of cross-sections of the mouse heart occurred in a diffuse manner, though there appeared to be some preference for the subendocardial and subepicardial regions at earlier time point of 15 minutes. No other organ showed uptake except kidney glomeruli, limited to the cortex, and rare lung capillaries, to a much lesser extent than heart tissue. Furthermore, CTP was able to transduce heart tissue *in vivo* far more efficiently and in a tissue-specific manner as compared to 8-Lysine, a known PTD (Fig. 7).

Since the initial description of *in vivo* screening of phage display libraries by Pasqualini and Ruoslahti [13], this approach has been utilized to identify peptides that target tumor vasculature [5], adipose tissue [6], pancreatic islet cells [7], synoviocytes [8], atherosclerotic plaques [9] as well as heart endothelial cells [10]. This approach also has been used in cell culture with adherent primary cardiomyocytes to isolate a 20-mer peptide with a homology to tenascin-X [11], an extracellular matrix protein. The phage displaying this peptide was found to be associated with cardiomyocytes isolated from mice treated with it *in vivo*. However, although it was preferentially associated with cardiomyocytes, it could still be isolated from lung tissue. Furthermore, it was unclear whether this 20-mer peptide was able to function as a cardiac transduction domain and transduce heart tissue *in vivo* independent of the phage carrying it.

In our screening approach, we combined a combination of cell culture and *in vivo* screening to identify a peptide able to be internalized into cardiac tissue. The first cycle was performed on cardiomyocytes as a screening approach to limit the population of non-specific phage from the initial phage library. All subsequent cycles were *in vivo* with intravenous injection in mice followed by a prolonged circulation time of 24 hours. Using this approach we identified a peptide that is able to deliver fluorescently labeled Streptavidin, a ∼60 kDa complex, to cardiac cells *in vivo* without transduction of liver, spleen, skeletal muscle or brain, with minimal uptake by lung and glomerular capillaries. A BLAST search in the NCBI data base did not reveal homology to any known, naturally occurring proteins.

Interestingly two separate groups of investigators, using an *in vitro* screening of a phage display library approach, have identified the exact same sequence as CTP, and shown it to have high affinity for binding to apatite-based, bone-like minerals [14] and two specific sulfated carbohydrates [15]. However, it is unclear how the ability of CTP to interact with these cellular components *in vitro* facilitates transduction of cardiac specific tissue *in vivo*. We currently are examining whether the transduction process is energy independent as well as whether it involves endocytosis. Also, the size limitation for the cargo to be delivered is unknown, but presumably it is as large as or larger than the M13 phage used for the identification of the peptide.

Given the fact that CTP-mediated transduction of cardiac tissue is efficient, specific and rapid, it could be used to deliver a variety of proteins, peptides, small molecules and viral and non-viral gene transfer vectors to the heart for treating cardiac conditions. In addition, it could be used diagnostically to determine the extent of viable cardiac tissue following infarct or ischemia reperfusion injury. Overall, the identification of a heart-specific delivery peptide should allow for novel biological treatments for cardiac conditions.
